# Supplementary material for: Drivers of Broad-Spectrum Antibiotic Overuse across Diverse Hospital Contexts—A Qualitative Study of Prescribers in the UK, Sri Lanka and South Africa
Source: Antibiotics (Basel). 2021 Jan 19;10(1):94. doi: 10.3390/antibiotics10010094 (PMC7835907; doi:10.3390/antibiotics10010094)
Supplement: Supplementary file 1 [file antibiotics-10-00094-s001.pdf]

Factors impacting on BSA overuse: Illustrative quotes

	**Illustrative quotes**
Individual factors	
Diagnostic uncertainty	*When doing the casualty, when the patient come in with low blood pressure but there is no obvious site of infection, and we are not sure whether it is septic shock and there may be underlying cardio. […] In some situations we tend to treat everything like to cover all aspects in that situations, sometimes we are going for broad spectrum antibiotic usage. (SL016, public hospital)*
Prioritising reduction of risk for individual patients	*You can have excellent activity or excellent cover from the very first point of antibiotic administration. And we know about the mortality of very sick patients in hospital, […] So broad-spectrum antibiotic decreases mortality. (SA010, public hospital)*
Fear of repercussions from not prescribing	*I want the patient to get better, and I want, I mean the thing is always, that would I be able to defend myself in retrospect. […] And I think litigation has probably contributed to abuse of antibiotics. (SA003, private hospital)*
Perceptions of BSAs as 'powerful' and effective	*So we give them these broad-spectrum and then they become well, just fit and well in two days and start responding after one or two doses. So when they came here they're crying, and after just a few hours they are fit and well. (UK013, public hospital)*
Concern about AMR as a pressing problem	*I don't think as clinicians we really think about resistance, […] on a daily basis I probably don't worry about it too much […]. And there's so many options out there, antibiotics are so common (UK008, public hospital)*
Training/knowledge/experience in antibiotic use	*I have not got any specific training on antibiotics. During the period where I learned microbiology and also during my medicine MD training, we used to go through the guidelines regarding certain diseases but I have not got any specific training. (SL016, public hospital)*
Engagement with antibiotic guidelines and policies	*I think we just follow the ones that come from [Organisation 1] basically, the usual ones that infection control and infectious diseases give out. […] I haven't looked at them for a long time. (SA007, private hospital)*
Social factors	
Social norms/culture of antibiotic use	*For instance in [ward] they would just put people on broad-spectrum antibiotics without proper like microbiological proof that the patient has this infection, or that this infection is causing the patient's clinical*

	picture.[…] I think some of the doctors are fixed in their ways, they have done this this way, and yeah, they don't realise that we actually have to start looking at our antibiotic choices. (SA011, public hospital)
Clinical autonomy	*You obviously do have colleagues which overuse different kinds of antibiotics, which is inappropriate. So in a private hospital like this, it's got a lot to do about individuality, certain people with certain preferences. (SA002, private hospital)*
Hierarchy and willingness to challenge colleagues	*IS THERE ANYBODY WHO INFLUENCES YOUR DECISION ON BROAD-SPECTRUM USE?*<br>*So consultants. So if my senior tells me to do it. I will try and challenge as much as possible, but at the end of the day, if it comes to a confrontation and it comes to a breakdown of relationship […], if it's a consultant, it is their responsibility to which antibiotic they think is appropriate. (UK004, public hospital)*
Structural factors	
Conflicting quality & safety agendas	*With the big drive for sepsis six coming in, it becomes even harder as an individual to say antibiotics aren't needed (UK006, public hospital)*
Pressure/incentives to satisfy patient demand	*P7, MC: If [patients] don't get [antibiotic] here, they will go to the next place and the next doctor will prescribe it and they will come back, and they will go 'You are a bad doctor'. (SA007, private hospital)*
Hospital environment	*P2, NE: And also regarding our hospital, we are having only a very poor infrastructure because the toilets are not good and there is no hot water. [...] Minimum facilities for the patients. [...] So considering antibiotics we have to consider this whole set-up. (SL002, public hospital)*
Limited microbiology facilities	*There's some investigations not available in our hospital […] lab facilities wise. […]*<br>*Microbiology, investigations for deciding what antibiotics. Sometimes they are not available. (SL002, public hospital)*
Level of strain on the system/need for efficiency	*Sometimes you have 20 patients in 20 minutes, the consultants are rushing, like Dr [name], my consultant, multiple jobs, so we don't have much time to spend on one patient and go through everything. (UK013, public hospital)*
Availability and quality of antibiotics in hospital	*To be honest we are not sure about the antibiotic quality of the narrow spectrum ones available. (SL015, public hospital)*

Patient poverty	*These patients are very poor patients, they don't have money to pay outside drugs, so we have to give all the drugs from hospital. So our hospital's available oral drugs are those oral Co-amoxiclav and oral Cefuroxime like things [BSAs]. […] We have to continue full course of antibiotics and we can't change the oral antibiotics. (SL004, public hospital)*
Delayed presentation by patients	*I get people coming from the countryside, that have been lying with appendicitis for two or three days already. And they have a septic abdomen. (SA004, private hospital)*
Poor/uncontrolled community healthcare resources	*The problem is most of the doctors practicing [in the community], they are prescribing these drugs, and patients getting one or two doses and then they stop the treatment, and then they are appearing in the hospital. (SL004, public hospital)*
